# Synergistic Activity of Carfilzomib and Panobinostat in Multiple Myeloma Cells via Modulation of ROS Generation and ERK1/2

**DOI:** 10.1155/2015/459052

**Published:** 2015-04-27

**Authors:** Lu Gao, Minjie Gao, Guang Yang, Yi Tao, Yuanyuan Kong, Ruixue Yang, Xiuqin Meng, Gongwen Ai, Rong Wei, Huiqun Wu, Xiaosong Wu, Jumei Shi

**Affiliations:** Department of Hematology, Shanghai Tenth People's Hospital, Tongji University School of Medicine, Shanghai 200072, China

## Abstract

Relapse of disease and subsequent resistance to established therapies remain as major challenges in the treatment of multiple myeloma (MM). New therapeutic options are needed for these extensively pretreated patients. To explore an optimized combinational therapy, interactions between the irreversible proteasome inhibitor carfilzomib exhibiting a well-tolerated side-effect profile and histone deacetylase inhibitor (HDACi) panobinostat (LBH589) were examined in MM cells. Coadministration of carfilzomib and LBH589 led to a synergistic inhibition of proliferation in MM cells. Further studies showed that the combined treatment synergistically increased mitochondrial injury, caspase activation, and apoptosis in MM cells. Lethality of the carfilzomib/LBH589 combination was associated with the reactive oxygen species (ROS) generation and ERK1/2 inactivation. In addition, the free radical scavenger N-acetylcysteine (NAC) could block carfilzomib and LBH589-induced oxidative stress and the subsequent apoptosis. Together, these findings argue that the strategy of combining carfilzomib and LBH589 warrants attention in MM.

## 1. Introduction

Multiple myeloma (MM) is a B-cell malignant disorder characterized by clonal proliferation of plasma cells in the bone marrow and osteolytic bone lesions [1]. Although new therapeutic options have been introduced and overall survival rate has improved in the management of MM, the disease remains incurable and almost all patients show disease relapse and develop drug resistance because of rapid regrowth of chemotherapy-refractory MM cells. This indicates that efficacious novel therapies are still needed for the patients with relapsed/refractory MM. Recently, data from present studies showed that the combination of proteasome inhibitor and histone deacetylase inhibitor (HDACi) resulted in synergistic inhibition of MM cell growth and might be an effective therapy for such patients [2, 3].

Carfilzomib, a second-generation selective, irreversible proteasome inhibitor of the chymotrypsin-like activity of the proteasome, shows antimyeloma effects [4]. Carfilzomib has been approved for the treatment of relapsed/refractory MM by the US Food and Drug Administration. It is well-tolerated in humans, especially a low incidence of peripheral neuropathy, and has activity against bortezomib-resistant myeloma cells, which makes it particularly suitable for use in combinational strategies [5]. Previous clinical trial has shown that replacing bortezomib with carfilzomib is safe and effective for MM patients failing bortezomib-containing combination regimens [6].

Panobinostat (LBH589), a highly potent HDACi, displays antitumor activity against a range of malignancies, particularly hematological diseases, such as MM, cutaneous T-cell lymphoma, Hodgkin lymphoma, and chronic myelogenous leukemia [7, 8]. LBH589 has shown activity against drug-resistant cancer cell. LBH589 in combination with other therapies has shown synergistic antitumor efficacy by preclinical studies [8]. Some phase I/II clinical trials have been conducted to investigate the safety and efficacy of LBH589 in combination with other agents [9–11].

Present studies have demonstrated that the proteasome inhibitor bortezomib/HDACi combination has a powerful antimyeloma activity on MM cells including cells that are highly resistant to cytotoxic drugs [12, 13]. Clinical trials further confirm such activity in relapsed/refractory MM patients [3, 9]. However, a considerable part of patients in these clinical trials could not tolerate such therapy because of serious side effects and discontinued treatment [9]. This constrains its application to some extent and an optimized proteasome inhibitor/HDACi combination with lesser side effects is therefore needed. The second-generation proteasome inhibitor carfilzomib has a well-tolerated side-effect profile and potent antimyeloma activity. Thus, the carfilzomib/HDACi combination may represent an optimized proteasome inhibitor/HDACi combination therapy for MM patients if synergistic interactions between them exist. The purpose of the present study is to determine whether the combination of carfilzomib and LBH589 could have a synergistic activity on MM cells. Our results indicate that carfilzomib and LBH589 interact in a highly synergistic manner in all four tested MM cells and that events involve triggering reactive oxygen species (ROS) generation and inhibiting ERK1/2 pathway. Thus, our research provides a basis for clinical evaluation of this possible optimized combination of proteasome inhibitor and HDACi in relapsed/refractory MM patients.

## 2. Materials and Methods

### 2.1. Cells

The human MM cell lines RPMI 8226, OPM2, U266, and H929 were purchased from Cell Resource Center of Shanghai Institutes for Biological Sciences (Shanghai, China). Cells were maintained in RPMI-1640 medium (Invitrogen, Frederick, USA) containing 10% fetal bovine serum, 1% penicillin (100 units/mL), and 1% streptomycin (100 mg/mL).

### 2.2. Reagents

Carfilzomib was purchased from Onyx Pharmaceuticals (South San Francisco, USA). LBH589 was purchased from Merck & Co., Inc. (Rahway, USA). These agents were dissolved in dimethyl sulfoxide. N-Acetylcysteine (NAC) was purchased from Sigma-Aldrich (St. Louis, USA) and prepared in double-distilled water before use. Cell Counting Kit-8 (CCK-8) was purchased from Dojindo (Mashikimachi, Japan). Cell apoptosis kit was obtained from BD Pharmingen (Franklin Lakes, USA). JC-1 Mitochondrial Membrane Potential Assay kit was from Beyotime Institute of Biotechnology (Haimen, China).

### 2.3. Cell Survival Assay

MM cells were seeded into 96-well plates at a density of 2 × 10^5^ cells per well and treated with different concentrations of carfilzomib and/or LBH589 for 48 h. CCK-8 was added into each well for an additional 2 h at 37°C. The optical density was measured at 450 nm using a microplate reader and the cell survival rate was expressed as the absorbance relative to that of controls.

### 2.4. Assessment of Cell Apoptosis

After different drug treatments, RPMI 8226 cells were stained with Annexin V (BD Pharmingen, Franklin Lakes, USA) and propidium iodide (PI) (BD Pharmingen, Franklin Lakes, USA) according to the manufacturer's instructions. In our studies, the early apoptotic cells displayed Annexin V^+^/PI^−^ staining and the late apoptotic cells displayed Annexin V^+^/PI^+^ staining.

### 2.5. Analysis of Mitochondrial Membrane Potential

The changes in mitochondrial membrane potential (ΔΨm) were measured by flow cytometry using JC-1 staining according to the manufacturer's instructions. Briefly, RPMI 8226 cells were stained with 1X JC-1 working solution for 20 min at 37°C. Then cells were washed with JC-1 staining buffer and analyzed by flow cytometry.

### 2.6. Western Blot Analysis

Cells were lysed in lysis buffer (100 mM Tris-HCl, pH 6.8, 4% SDS, 20% glycerol) on ice for 30 min. Proteins (30 *μ*g) were subjected to 10% or 12% SDS-PAGE and transferred to nitrocellulose membrane. The membranes were blocked with 5% bovine serum albumin for 1 h and probed with primary antibodies overnight at 4°C, followed by treatment with appropriate secondary antibodies. Primary antibodies were as follows: caspase-9, cleaved caspase-8, cleaved caspase-3, phospho-p44/42 MAPK (phospho-ERK1/2), p44/42 MAPK (ERK1/2), p38 mitogen-activated protein kinase, phospho-p38 mitogen-activated protein kinase, and *β*-actin antibodies. All were from Cell Signaling Technology (Beverly, USA).

### 2.7. Cell Cycle Distribution Analysis

Cells were collected and washed with ice cold phosphate buffered saline (PBS), fixed in 75% ethanol at −20°C for 16 h, and stained at 37°C for 15 min with PI containing 50 mg/mL RNase (BD Pharmingen, Franklin Lakes, USA) followed by flow cytometric analysis.

### 2.8. Measurement of ROS Generation

Cells were pretreated with or without NAC for 2 h at 37°C and then incubated with various drugs for indicated times. Then the cells were washed with PBS, resuspended in RPMI-1640 medium containing 10 *μ*M of 2′,7′-dichlorodihydrofluorescein diacetate (DCFH-DA) (Beyotime, Haimen, China), and incubated at 37°C for 20 min. Fluorescence intensity was assessed using a flow cytometer (BD, San Diego, USA).

### 2.9. Statistical Analysis

All data were expressed as mean ± standard deviation (SD). Statistical significance of differences in multiple comparisons was determined by one-way ANOVA. *P* < 0.05 was considered significant. Combination index (CI) was calculated using median dose effect analysis in conjunction with a commercially available software program (CalcuSyn, Biosoft).

## 3. Results

### 3.1. Concomitant Treatment with Carfilzomib and LBH589 Results in a Synergistic Inhibition of MM Cells Survival* In Vitro*

**Figure 1:**
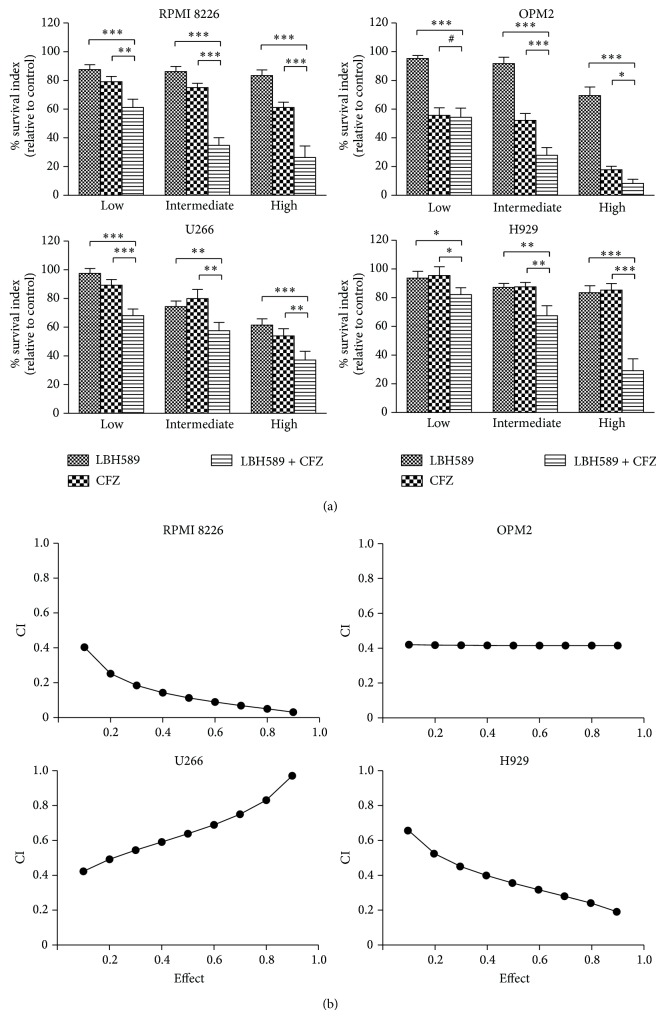
Coadministration of carfilzomib and LBH589 induced a synergistic inhibition of proliferation in MM cells. (a) All cell lines were incubated for 48 h with carfilzomib and/or LBH589 (low: 2 nM LBH589, 20 nM carfilzomib; intermediate: 4 nM LBH589, 40 nM carfilzomib; and high: 6 nM LBH589, 60 nM carfilzomib) followed by CCK-8 assay. Data represent the mean ± SD for three separate experiments performed in triplicate. ^∗^ *P* < 0.05. ^∗∗^ *P* < 0.01. ^∗∗∗^ *P* < 0.001. ^#^ *P* > 0.05. CFZ, carfilzomib. (b) CI values were calculated using median dose effect analysis. CI values < 1.0 denote synergistic interactions.

To assess what effect the combination of carfilzomib and LBH589 would have on MM cell survival, RPMI 8226, OPM2, U266, and H929 cells were incubated with increasing concentrations of carfilzomib and/or LBH589 for 48 h, after which cytotoxicity was evaluated by CCK-8 assay. As shown in Figure 1(a), compared to individual exposure (excepting individual exposure of OPM2 cells to low concentration of carfilzomib), combined exposure to low, intermediate, and high concentrations of carfilzomib and LBH589 induced a more significant decrease in the growth of all four tested MM cell lines (*P* < 0.05). The median dose effect analysis for all tested MM cells exposed to carfilzomib and LBH589 yields CI values which were substantially less than 1.0, indicating a synergistic interaction (Figure 1(b)).

### 3.2. Concomitant Treatment with Carfilzomib and LBH589 Effectively Induces Apoptosis, Mitochondrial Injury, and Caspase Activation in MM Cells

**Figure 2:**
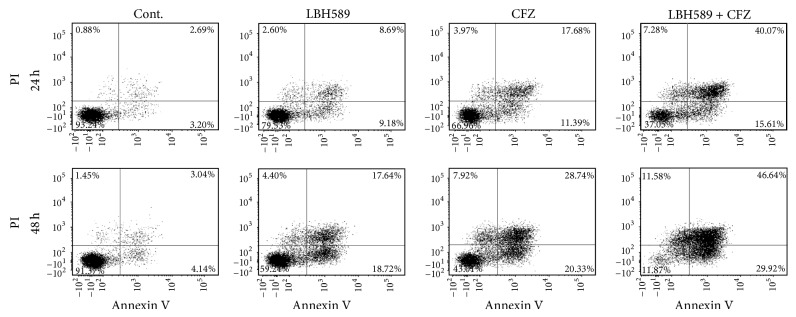
Combination of carfilzomib and LBH589 induced enhanced apoptosis in MM cells. RPMI 8226 cells were exposed to carfilzomib (40 nM) and/or LBH589 (4 nM) for 24 h (upper panel) or 48 h (low panel) followed by Annexin V-FITC/PI double staining and flow cytometry analysis. The percentage of apoptotic cells (24 h) in Cont., LBH589, CFZ, and LBH589 + CFZ group was 6.7% ± 0.9%, 18.8% ± 2.8%, 30.0% ± 3.5%, and 56.6% ± 8.8%^∗^, respectively. The value (48 h) was 6.8% ± 1.5%, 35.9% ± 3.8%, 48.3% ± 3.8%, and 81.5% ± 5.0%^∗^, respectively. ^∗^ *P* < 0.05 versus control group; *n* = 3. The data shown are representative of three independent experiments. Cont., control. CFZ, carfilzomib.

Annexin V/PI double staining was performed to determine the apoptosis of RPMI 8226 cells exposed to carfilzomib (40 nM) and/or LBH589 (4 nM) for 24 h or 48 h. Compared with the control, individual treatment with LBH589 only caused a moderate increase in the percentage of Annexin V^+^ cells (18.8% ± 2.8% versus 6.7% ± 0.9% for 24 h and 35.9% ± 3.8% versus 6.8% ± 1.5% for 48 h). The values were relatively high in carfilzomib treated RPMI 8226 cells (30.0% ± 3.5% for 24 h and 48.3% ± 3.8% for 48 h). On the other hand, MM cells exposed to carfilzomib and LBH589 exhibited a much higher percentage of Annexin V^+^ cells (56.6% ± 8.8% for 24 h and 81.5% ± 5.0% for 48 h), indicating that the combination resulted in a significant induction of apoptosis (Figure 2).

**Figure 3:**
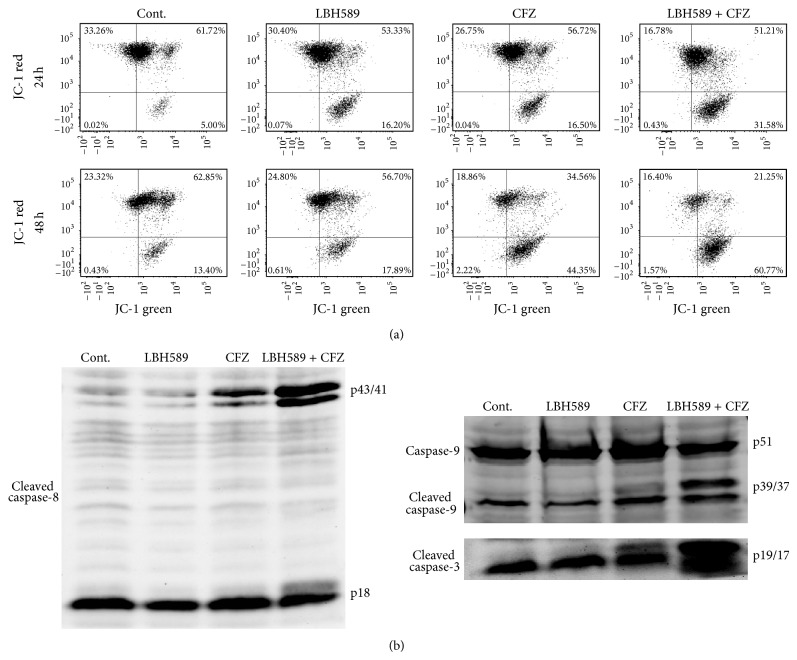
Concomitant treatment with carfilzomib and LBH589 synergistically resulted in mitochondrial injury and caspase activation. (a) RPMI 8226 cells were treated with carfilzomib (40 nM) and/or LBH589 (4 nM) for 24 h (upper panel) or 48 h (low panel), after which JC-1 staining was performed. ΔΨm was assessed by flow cytometry. Only JC-1 green positive (lower right quadrant) cells were analyzed for the loss of ΔΨm. The loss of ΔΨm (24 h) in Cont., LBH589, CFZ, and LBH589 + CFZ group was 5.3% ± 2.1%, 16.3% ± 2.9%, 20.3% ± 4.0%, and 35.0% ± 3.0%^∗∗∗^, respectively. The value (48 h) was 12.2% ± 3.5%, 20.8% ± 3.1%, 40.1% ± 4.6%, and 62.4% ± 5.0%^∗∗∗^, respectively. ^∗∗∗^ *P* < 0.001 versus control group; *n* = 3. (b) RPMI 8226 cells were treated with carfilzomib (40 nM) and/or LBH589 (4 nM) for 24 h. Then, caspase-9 (p51) and cleaved caspase-8 (p43/41, p18), caspase-9 (p39/37), and caspase-3 (p19/17) were monitored by Western blot analysis. Cont., control. CFZ, carfilzomib.

The reduction of ΔΨm is a vital event in the initiation of apoptotic cascade. The combined treatment of carfilzomib (40 nM) and LBH589 (4 nM) induced 35.0% ± 3.0% loss of ΔΨm in RPMI 8226 cells at 24 h, as represented by the cells with decreased JC-1 red fluorescence, whereas the loss of ΔΨm was only 20.3% ± 4.0% for carfilzomib (40 nM) and 16.3% ± 2.9% for LBH589 (4 nM) at 24 h. Moreover, a more significant loss of ΔΨm was observed in the combinational treatment (62.4% ± 5.0%) compared with those treated with carfilzomib (40.1% ± 4.6%) or LBH589 (20.8% ± 3.1%) alone at 48 h (Figure 3(a)). To further confirm that the combined treatment with carfilzomib and LBH589 did trigger classical apoptosis in MM cells, caspase activation, another pivotal event associated with the activation of apoptotic cell death, was examined by Western blot analysis. As shown in Figure 3(b), a clear cleavage of caspase-8, caspase-9, and caspase-3 was observed in RPMI 8226 cells after incubation with both carfilzomib (40 nM) and LBH589 (4 nM) for 24 h. In contrast, only modest cleavage of all three caspase proteins was detected in RPMI 8226 cells after 24-hour treatment of carfilzomib (40 nM) or LBH589 (4 nM) alone. These findings indicate that combined treatment of MM cells with carfilzomib and HDACi LBH589 potently induces ΔΨm loss and caspase activation, events associated with activation of the apoptotic program.

### 3.3. The Effects of Carfilzomib and/or LBH589 on Cell Cycle Distribution

**Figure 4:**
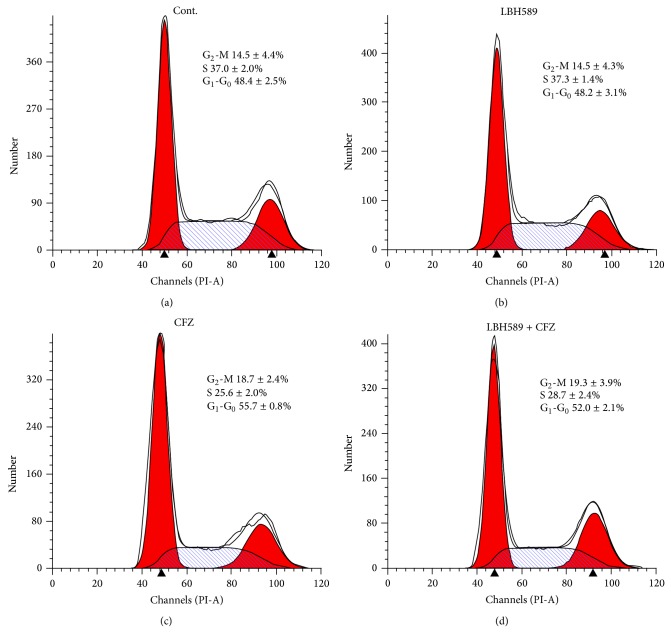
Concomitant treatment with carfilzomib and LBH589 showed no effect on MM cell cycle distribution. MM cells were treated with carfilzomib (40 nM) and/or LBH589 (4 nM) for 24 h followed by PI staining and flow cytometry analysis. Data represent the mean ± SD for three separate experiments performed in triplicate. Only the percentage of G_1_-G_0_ in CFZ group was significantly different from that in control group (*P* < 0.01). The percentage of G_1_-G_0_ in LBH589 and LBH589 + CFZ group and G_2_-M in CFZ, LBH589, and LBH589 + CFZ group was similar to that in control group (*P* > 0.05). Cont., control. CFZ, carfilzomib.

Cell cycle analysis was performed in U266 cells exposed to carfilzomib (40 nM) and/or LBH589 (4 nM) for 24 h. Compared with the control, treatment with carfilzomib resulted in G_1_-G_0_ arrest accompanied by a decrease in S phase cell population (*n* = 3, *P* < 0.01). Individual treatment with LBH589 had little effect on cell cycle distribution. Similarly, neither G_1_-G_0_ arrest nor G_2_-M arrest was observed in combined treatment (*n* = 3, *P* > 0.05) despite the synergistic induction of apoptosis caused by combined treatment (Figure 4). Similar results were observed in RPMI 8226 cells after treatment with carfilzomib (40 nM) and/or LBH589 (4 nM) for 24 h (data not shown).

### 3.4. Combined Exposure of MM Cells to Carfilzomib and LBH589 Induces ROS Generation

**Figure 5:**
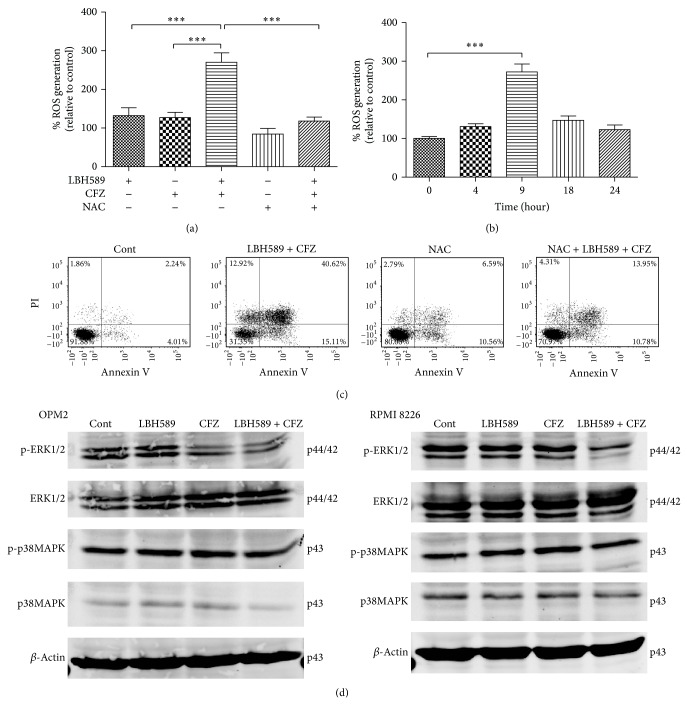
Lethality of the carfilzomib/LBH589 combination in MM cells was associated with the ROS generation and ERK1/2 inactivation. (a) RPMI 8226 cells were pretreated with or without NAC for 2 h at 37°C and then incubated with carfilzomib (40 nM) and/or LBH589 (4 nM) for 24 h, after which ROS generation was detected. ^∗∗∗^ *P* < 0.001. *n* = 3. (b) ROS generation in RPMI 8226 cells was monitored 0, 4, 9, 18, and 24 hours after combined treatment with carfilzomib (40 nM) and LBH589 (4 nM). ^∗∗∗^ *P* < 0.001. *n* = 3. (c) RPMI 8226 cells were pretreated with or without NAC for 2 h at 37°C and then incubated with or without carfilzomib (40 nM) and LBH589 (4 nM) for 24 h. Apoptosis rate in Cont., LBH589 + CFZ, NAC, and NAC + LBH589 + CFZ group was 6.5% ± 1.9%, 55.3% ± 9.2%, 12.4% ± 4.2%, and 29.2% ± 4.3%^∗∗∗^, respectively. ^∗∗∗^ *P* < 0.001 versus LBH589 + CFZ group. *n* = 3. The data shown are representative of three independent experiments. (d) OPM2 and RPMI 8226 cells were treated with carfilzomib (40 nM) and/or LBH589 (4 nM) for 24 h. Then, Western blot analysis of ERK1/2, p-ERK1/2, p38MAPK, and p-p38MAPK was performed. The levels of *β*-actin were used as the loading control. Cont., control. CFZ, carfilzomib.

Previous studies have reported that cytotoxicity induced by bortezomib/HDACi combination originates from ROS generation [2, 14]. Studies were therefore performed to investigate whether such mechanism is also responsible for carfilzomib and LBH589-induced cytotoxicity. As shown in Figure 5(a), individual treatment with carfilzomib (40 nM) or LBH589 (4 nM) had modest effect on ROS levels in RPMI 8226 cells, whereas the combined treatment resulted in a marked increase in ROS generation, which was substantially abrogated by the free radical scavenger NAC (15 mM). In addition, 9 hours of combined treatment with carfilzomib (40 nM) and LBH589 (4 nM) induced the most obvious ROS generation in RPMI 8226 cells (Figure 5(b)). To assess the importance of ROS generation in carfilzomib and LBH589-induced apoptosis, RPMI 8226 cells were preincubated with NAC for 2 h and then treated with carfilzomib (40 nM) and LBH589 (4 nM) for 24 h. As expected, NAC significantly (*P* < 0.001) reduced the apoptosis induced by the combined treatment (Figure 5(c)), suggesting that ROS generation plays an important role in carfilzomib and LBH589-mediated cytotoxicity in MM cells.

### 3.5. Synergistic Induction of Apoptosis after Combined Treatment Involves the ERK1/2 Pathway

To determine the molecular mechanisms underlying the carfilzomib/LBH589 lethality in MM cells, several relevant signaling pathways were investigated. As shown in Figure 5(d), compared with the control, exposure of RPMI 8226 cells to carfilzomib (40 nM) and LBH589 (4 nM) for 24 h markedly decreased the level of ERK1/2 phosphorylation without obvious changes in the total ERK levels, suggesting that the ERK1/2 pathway, protecting MM cells from apoptosis, was effectively inhibited by the combined treatment. Carfilzomib (40 nM) or LBH589 (4 nM) alone had no significant effect on the phosphorylation of ERK1/2 and total ERK levels. A similar profile was observed in OPM2 cells. In the present study, p38MAPK signaling pathway, another major mechanism involved in the modulation of MM cell apoptosis, was also analyzed. As shown in Figure 5(d), the levels of total p38 and the p38 phosphorylation were much alike amongst different treatments.

## 4. Discussion

Combinational therapies, with agents that are synergistic when combined, are often required for patients with relapsed and/or refractory MM [15]. Among them, bortezomib/HDACi combination attracts the most attention because of powerful antimyeloma activity. However, side effects of bortezomib/HDACi combination observed in clinical trials confine its application in some MM patients [9]. In the present study, we examine the interactions between carfilzomib and HDACi LBH589 to explore a possible optimized combinational therapy of proteasome inhibitor and HDACi for MM.

We observed a synergistic inhibition of cell proliferation and apoptosis in MM cells after combined treatment with carfilzomib and LBH589. To further confirm the apoptosis, ΔΨm loss and caspase cleavage, events associated with apoptosis activation, were investigated. Our data showed that the levels of cleaved caspase-9, caspase-8, and caspase-3 were markedly increased in MM cells exposed to carfilzomib and LBH589 compared with single drug treatment, suggesting that both intrinsic (caspase-9) and extrinsic (caspase-8) apoptotic pathways were activated after combined treatment. Moreover, the more loss of ΔΨm in both drugs treated cells further indicated the activation of intrinsic apoptotic pathway.

Since cell cycle arrest is often associated with apoptosis and several studies have shown that the proteasome inhibitor/HDACi combination, inducing obvious apoptosis, caused G_1_-G_0_ or G_2_-M arrest [13, 14], cell cycle analysis was performed in the present study. Our data showed that carfilzomib alone induced G_1_-G_0_ arrest in MM cells, whereas no significant changes in cell cycle distribution were observed after individual treatment with LBH589 or combined treatment. The result was consistent with another study in which the proteasome inhibitor/HDACi combination also failed to induce G_1_-G_0_ or G_2_-M arrest [16]. Reasons for the discrepancy in these studies are unclear, but variations in cell types and different doses of the combined drugs may be responsible for the observed difference.

Previous studies in various tumor cells have indicated that bortezomib or HDACi-induced lethality is related to ROS generation [17–20]. Moreover, lethal effects induced by the combined treatment with proteasome inhibitor and HDACi in leukemia and lymphoma cells have also been demonstrated to proceed through a ROS-dependent mechanism [21, 22]. In the present study, we showed that combined treatment with carfilzomib and LBH589 induced a marked increase in ROS in MM cells and the free radical scavenger NAC attenuated the oxidative stress, as well as the subsequent apoptosis. Thus, our data provide further support for the notion that ROS generation is a crucial factor in proteasome inhibitor/HDACi-mediated lethality.

Activation of ERK1/2 pathway has been shown to protect malignant cells from the lethality of oxidative stress [23] and therefore confers a survival advantage on these cells. Studies have indicated that apoptosis induction of several antitumor drugs, alone or in combination, is associated with inactivation of this cytoprotective pathway. For example, inhibition of ERK1/2 pathway is one of the molecular mechanisms underlying lethality of bortezomib and HDACi combination on T-leukemia/lymphoma cells [24]. In our present study, when carfilzomib was combined with LBH589, a significant decrease in the level of ERK1/2 phosphorylation was observed, whereas phosphorylation of p38 exhibited no change. This suggests that inhibition of the ERK1/2 pathway may be the right mechanism through which carfilzomib and LBH589 combination induces apoptosis.

In summary, our data indicate that carfilzomib and LBH589 combination synergistically induces apoptosis in MM cells, which is accomplished by enhancing ROS generation and decreasing ERK1/2 phosphorylation. Thus, we provide a basis for clinical evaluation of carfilzomib/LBH589 combination in relapsed/refractory MM patients.
